# Anti-Nucleocapsid Protein Immune Responses Counteract Pathogenic Effects of Rift Valley Fever Virus Infection in Mice

**DOI:** 10.1371/journal.pone.0025027

**Published:** 2011-09-16

**Authors:** Petrus Jansen van Vuren, Caroline T. Tiemessen, Janusz T. Paweska

**Affiliations:** 1 Special Pathogens Unit, National Institute for Communicable Diseases of the National Health Laboratory Service, Sandringham, South Africa; 2 Division Virology and Communicable Diseases Surveillance, School of Pathology, University of the Witwatersrand, Johannesburg, South Africa; 3 Cell Biology/AIDS Virus Research Unit, National Institute for Communicable Diseases of the National Health Laboratory Service, Sandringham, South Africa; University of Texas Medical Branch, United States of America

## Abstract

The known virulence factor of Rift Valley fever virus (RVFV), the NSs protein, counteracts the antiviral effects of the type I interferon response. In this study we evaluated the expression of several genes in the liver and spleen involved in innate and adaptive immunity of mice immunized with a RVFV recombinant nucleocapsid protein (recNP) combined with Alhydrogel adjuvant and control animals after challenge with wild type RVFV. Mice immunized with recNP elicited an earlier IFNβ response after challenge compared to non-immunized controls. In the acute phase of liver infection in non-immunized mice there was a massive upregulation of type I and II interferon, accompanied by high viral titers, and the up- and downregulation of several genes involved in the activation of B- and T-cells, indicating that both humoral and cellular immunity is modulated during RVFV infection. Various genes involved in pro-inflammatory responses and with pro-apoptotic effects were strongly upregulated and anti-apoptotic genes were downregulated in liver of non-immunized mice. Expression of many genes involved in B- and T-cell immunity were downregulated in spleen of non-immunized mice but normal in immunized mice. A strong bias towards apoptosis and inflammation in non-immunized mice at an acute stage of liver infection associated with suppression of several genes involved in activation of humoral and cellular immunity in spleen, suggests that RVFV evades the host immune response in more ways than only by inhibition of type I interferon, and that immunopathology of the liver plays a crucial role in RVF disease progression.

## Introduction

Rift Valley fever virus (RVFV), a mosquito borne *Phlebovirus* of the *Bunyaviridae* family, causes irregular but large outbreaks characterized by high fatality rates in young domestic ruminants and abortion storms in pregnant animals [1]–[3]. The RVFV RNA genome consists of large (L) and medium (M) segments which are in the negative sense, and a small (S) segment utilizing an ambisense coding strategy. The L segment encodes a viral RNA-dependent RNA polymerase, the M segment encodes two structural glycoproteins, two non-structural proteins, and the S segment encodes the nucleocapsid protein (NP) and a non-structural protein (NSs) [4], [5]. The NP is highly conserved and constitutes the most immunodominant viral protein in the *Bunyaviridae* family [6]–[11]. The NP associates with the viral RNA-dependent RNA polymerase and viral RNA to form the ribonucleoproteins (RNP) necessary for transcription and replication. The known virulence factor of RVFV, the NSs protein, counteracts the antiviral effects of the type I interferon response. Not much is known, however, about other molecular aspects of RVFV pathogenicity. Although the nucleocapsid protein (NP) of the virus does not induce production of neutralizing antibodies, anti-NP antibodies have been shown to be protective in mice [12]–[17]. Humoral responses against the RVFV glycoproteins effectively neutralize the virus and are believed to solely protect against challenge [14], [16], [18]–[20].

RVFV is sensitive to the actions of type I interferons [21]–[24] but it has developed several counteracting mechanisms. The NSs protein of RVFV interacts with a repressor complex (Sin3A/NCoR/HDAC) to inhibit transcriptional activation of the IFNβ gene [25], [26]. A more general shutdown of cellular gene expression is caused by interaction of NSs with the p44 subunit of the basal transcription factor II H (TFIIH), resulting in reduced transcriptional activity in RVFV infected cells [27]. The NSs has also been shown to act on a post-translational level by degrading Protein Kinase R (PKR) [28]. Interferon gamma (IFNγ) is a strong immunoregulator and has direct antiviral properties, but its role in the protection of the host against RVFV infection is debatable [29]. A study in rhesus monkeys showed that prophylactic treatment with recombinant human IFNγ before RVFV infection protected monkeys from clinical disease and decreased viremia significantly [30]. However, there was no marked difference in pathogenicity of RVFV MP-12 or Clone-13 in wild type mice compared to mice deficient in IFNγ receptor (IFNGR^−/−^), suggesting that NSs does not have the same inhibitory effect on the type II interferon response (IFNγ) as it has on the type I interferon response [31]. The RVFV M segment non-structural protein (NSm) was implicated in the pathogenesis of RVF by acting as an anti-apoptotic protein [32].

Do Valle et al. [33] utilizing microarray and quantitative PCR showed that a specific strain of mice, BALB/cByJ, was more resistant to RVFV infection when compared to a wild mouse strain, MBT/Pas. The study analyzed the expression of genes involved in the innate immune response by infecting mouse embryonic fibroblasts (MEF) isolated from both strains with RVFV *in vitro*, with results indicating a more significant type I IFN response in the BALB/cByJ MEFs.

The activation of adaptive immunity after RVFV infection at gene expression level in the main target organ for RVFV replication, the liver, as well as in the spleen which plays a very important role in host immunity, has not been investigated. A better understanding of the activation of memory humoral and cellular immune responses in these organs might aid the development of improved RVFV vaccines and identification of genes and their products as useful targets for development of gene therapy and antivirals. The role that anti-NP responses play in the protection of vaccinated individuals against RVFV infection is also not clearly understood.

The results from this study suggest that an earlier type I interferon response in recNP/Alhydrogel immunized mice contribute to decreased challenge virus replication, whereas upregulation of genes with the ability to result in immunopathology, combined with uncontrolled challenge virus replication, in non-immunized mice probably contributed to liver damage and morbidity, and downregulation of humoral and cellular immunity in the spleen possibly contributed to immune evasion.

## Results

### Immune response elicited by recNP/Alhydrogel immunization

**Figure 1:**
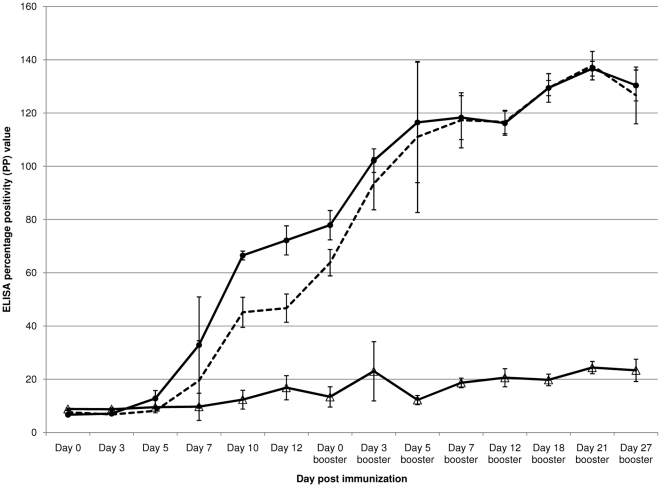
Anti-RVFV recNP response in recNP/Alhydrogel immunized mice. The total IgG (–•–), IgG1 subclass (—) and IgG2A subclass (–Δ–) responses are shown, with error bars indicating the standard deviations from the mean PP values of three biological replicates per time point.

The activation of either Th-1 cellular immunity, indicated by IgG2A subclass expression, or Th-2 humoral immunity, indicated by IgG1 subclass expression, was evaluated by testing serial bleeds after recNP/Alhydrogel immunization. Strong total IgG and IgG1 subclass responses were detectable by day 7 after the first immunization, with strong responses still detectable on the day before the booster inoculation (day 12) and RVFV challenge (day 27 after the booster)(IgG: average = 72.2 PP, standard deviation = 5.5 PP, OD_405 nm_ = 0.79; IgG1: average = 46.7 PP, standard deviation = 5.3 PP, OD_405 nm_ = 0.47 on day 12 after the initial immunization; IgG: average = 129.9 PP, standard deviation = 4.7 PP, OD_405 nm_ = 1.42; IgG1: average = 131.3 PP, standard deviation = 12.0 PP, OD_405 nm_ = 1.31 on day 27 after the booster, Figure 1). The IgG2A subclass response (IgG2A: average = 16.8 PP, standard deviation = 4.5 PP, OD_405 nm_ = 0.16) was weak compared to IgG1 and only detectable on day 12 after the first immunization (PP ratio of IgG1∶IgG2A = 2.8∶1). On the day before RVFV challenge (IgG2A: average = 23.4 PP, standard deviation = 4.2 PP, OD_405 nm_ = 0.22), a weak IgG2A response was still detectable(PP ratio of IgG1∶IgG2A = 5.6∶1) (Figure 1).

### Dynamics of gene expression and virus load in livers and spleens of immunized and control mice

**Figure 2:**
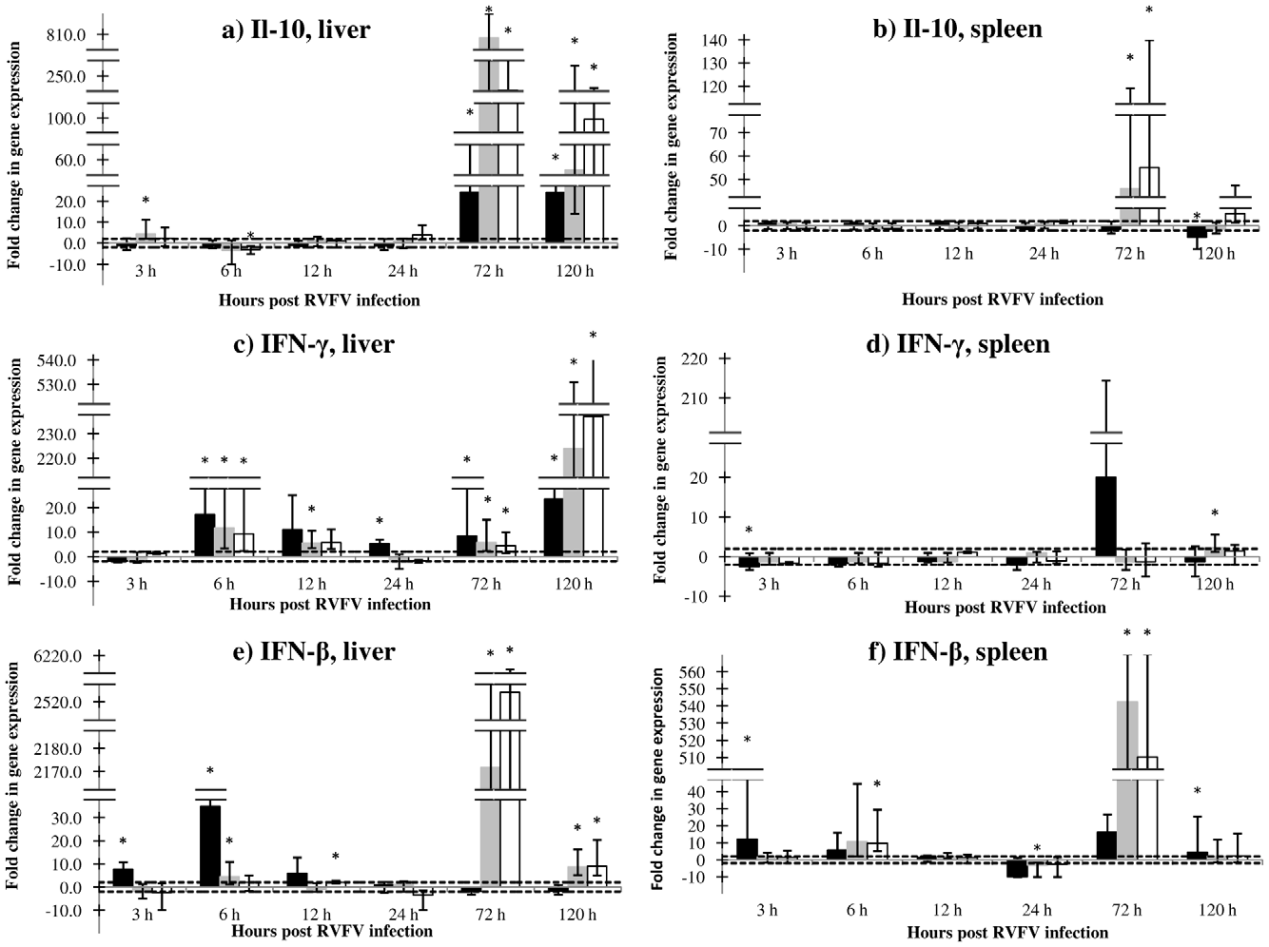
(a–f). Fold changes in expression of IL10, IFNγ and IFNβ genes in tissues of mice after RVFV infection. RecNP immunized mice (n = 3 per time point) are indicated by solid black bars, adjuvant control mice (n = 3 per time point) by grey bars and PBS control mice (n = 3 per time point) by white bars. The horizontal dotted lines indicate the cut-off values for upregulation (+2) or downregulation (−2). The asterisk (*) indicates where the *P*-value is smaller than or equal to 0.05 (statistically significant results). Standard error values are indicated by the error bars. Note the differences in the Y-axis scales. The following time points are indicated: 3, 6, 12, 24, 72 and 120 hours.

The normalized expression of IL-10, IFNγ and IFNβ was analyzed at 3, 6, 12, 24, 72 and 120 hours post RVFV infection in liver and spleen tissue of recNP immunized, and non-immunized adjuvant and PBS control mice. The results are shown in Figure 2a–f.

The expression of IL-10 was upregulated with statistical significance (4.5 fold, p<0.01) in adjuvant control mice but normal in recNP immunized mice at 3 hours p.i. in the liver (Figure 2a). At 72 and 120 hours p.i., however, IL-10 expression was massively upregulated in the adjuvant (72 hours, 808.3 fold, p = 0.033; 120 hours, 55.0 fold, p<0.01) and PBS control groups (72 hours, 243.3 fold, p<0.01; 120 hours, 99.3 fold, p = 0.034), and upregulated to a lesser extent in immunized mice (72 hours, 24.4 fold, p<0.01; 120 hours, 24.3 fold, p = 0.033) in the liver (Figure 2a). In the spleen expression of IL-10 was only upregulated significantly at 72 hours p.i. in control mice (adjuvant 46.0 fold, p<0.01; PBS 55.0 fold, p<0.01) (Figure 2b).

The expression of IFNγ was normal in all mice livers at 3 hours, but upregulated in all groups at 6 hours (recNP immunized, 17.2 fold, p<0.01; adjuvant control, 11.7 fold, p<0.01; PBS control, 9.3 fold, p<0.01), 72 hours (recNP immunized, 8.4 fold, p = 0.03; adjuvant control, 5.8 fold, p = 0.03; PBS control, 4.4 fold, p = 0.03) and 120 hours p.i. (recNP immunized, 23.4 fold, p = 0.03; adjuvant control 224.0 fold, p<0.01; PBS control 237.0 fold, p = 0.03) (Figure 2c). Expression of IFNγ remained unaltered in spleen tissue (Figure 2d).

At 3 hours post infection (p.i.) the expression of the IFNβ gene was significantly upregulated in the livers (7.7 fold, p = 0.03) and spleens (12.0 fold, p = 0.03) of recNP immunized mice, whereas expression was normal in adjuvant control and PBS control groups (Figure 2e–f). Expression of the gene remained upregulated in liver of immunized mice until 12 hours p.i., after which it waned (Figure 2e–f). In adjuvant control mice, IFNβ was massively upregulated at 72 hours p.i. in liver (2171.7 fold, p = 0.03) and spleen (542.3 fold, p<0.01), with similar results obtained in PBS control mice liver (2524.1 fold, p = 0.02) and spleen (510.3 fold, p<0.01) (Figure 2e–f). At 120 hours p.i. IFNβ expression remained upregulated in control mice liver.

**Figure 3:**
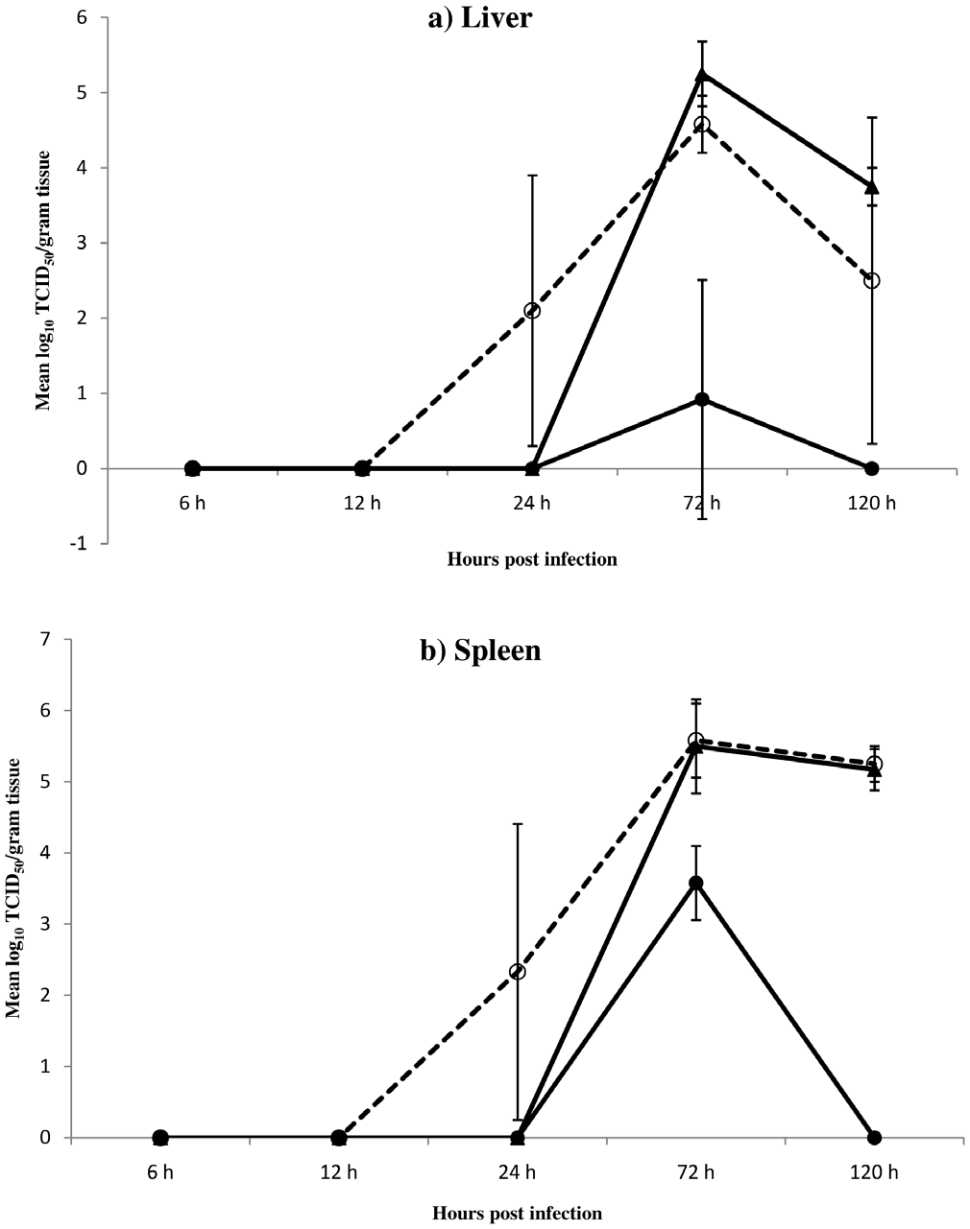
(a–b). Mean viral loads in livers and spleens of RVFV infected mice. Groups, consisting of 3 mice per group per time point, are indicated as: RecNP immunized (–•–), adjuvant control (–o–) and PBS control mice (–▴–). Livers are indicated in panel a, and spleens in panel b.

The challenge virus was first detected in the tissues of adjuvant control mice at 24 hours p.i. and, subsequently, at high titers in adjuvant and PBS controls at 72 and 120 hours p.i. Low titer of the virus was detected in liver of recNP-immunized mice only at 72 hours p.i. (10,000 fold lower than control mice). Surprisingly the virus replicated to relatively higher titers in the spleen of recNP-immunized mice, but only at 72 hours and still 100 fold lower compared to control mice (Figure 3a–b).

### The expression of genes involved in B- and T-cell immunity and other immune functions at 72 hours

The expression of an array of 84 genes involved in B- and T-cell immunity, and other immune functions, were analyzed in the liver and spleen tissues of recNP-immunized, and non-immunized adjuvant and PBS control mice at 72 hours post infection relative to an age related normal control group. The list of genes tested, fold change values for different groups and *P*-values are shown in Table S1.

**Figure 4:**
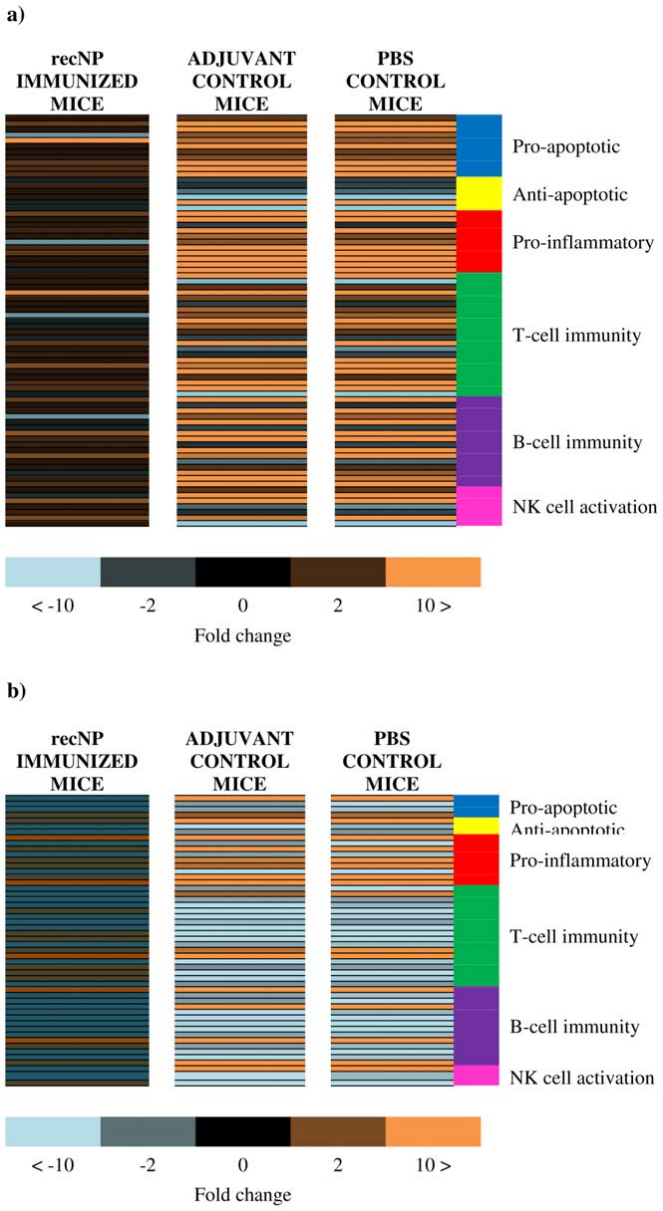
(a–b). Heat maps showing fold changes in liver and spleen at 72 hours after RVFV infection. Expression of genes in RecNP immunized, adjuvant control and PBS control mice are organized according to function. Livers are indicated in panel a, and spleens in panel b. The genes shown in orange are upregulated, those in blue are downregulated and those in black or darker shades of orange and blue have fold-change values between −2 and 2 and/or have p-values>0.05.

**Figure 5:**
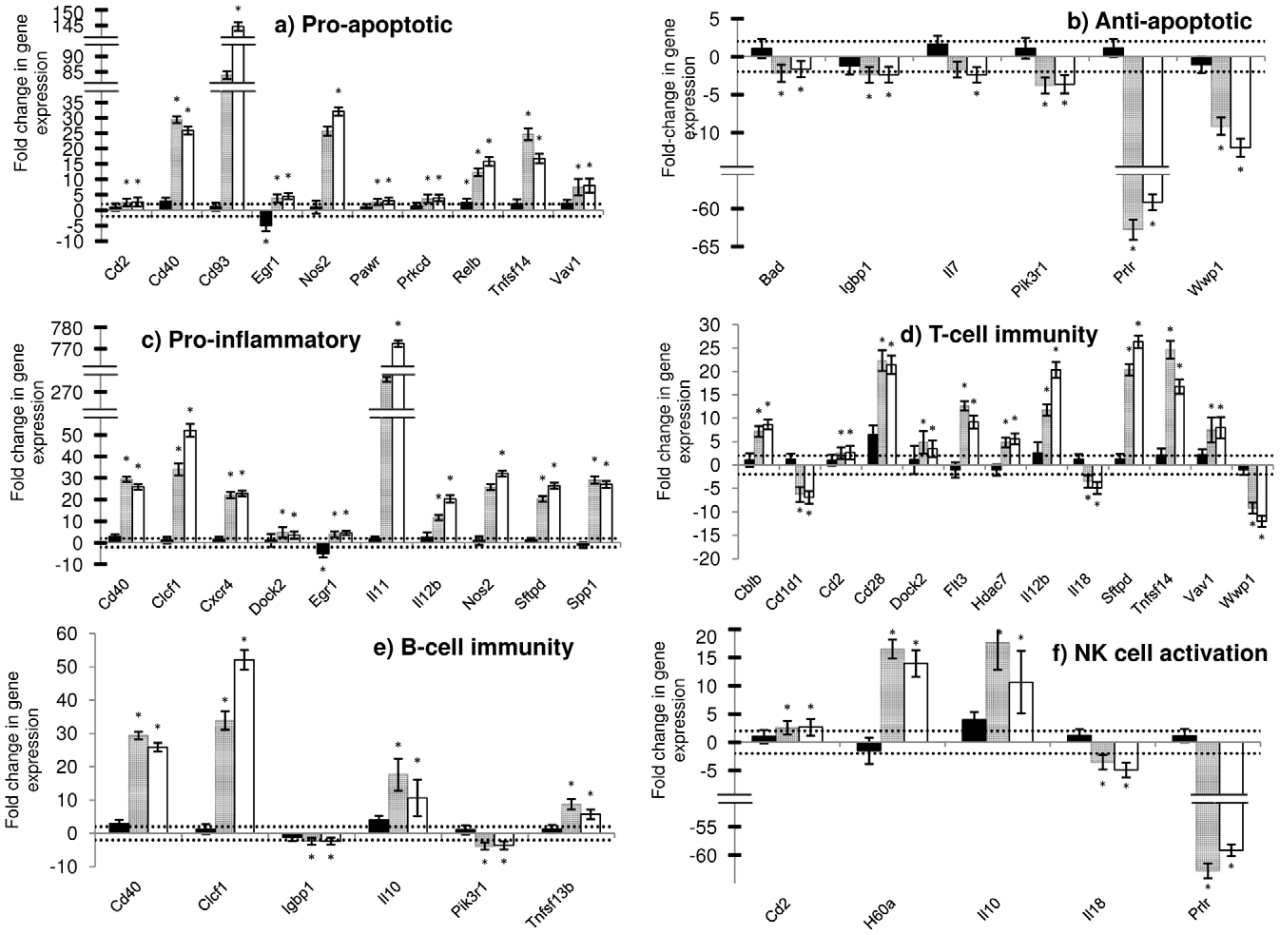
(a–f). Fold changes in expression of genes in the liver of experimental groups at 72 hours after RVFV infection. RecNP-immunized mice (n = 3) are indicated by solid black bars, adjuvant control mice (n = 3) by grey bars and PBS control mice (n = 3) by white bars. The horizontal dotted lines indicate the cut-off values for upregulation (+2) or downregulation (−2). The asterisk (*) indicates where the *P-*value is smaller than or equal to 0.05 (statistically significant results). Standard deviation from the mean fold changes are indicated by the error bars.

**Figure 6:**
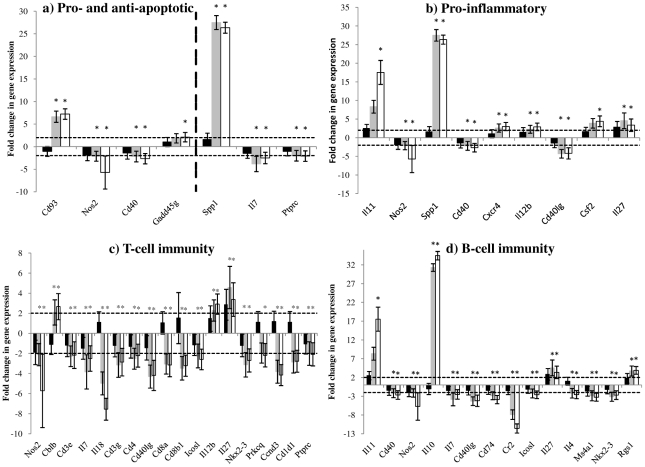
(a–d). Fold changes in expression of genes in the spleen of experimental groups at 72 hours after RVFV infection. RecNP-immunized mice (n = 3) are indicated by solid black bars, adjuvant control mice (n = 3) by grey bars and PBS control mice (n = 3) by white bars. The horizontal dotted lines indicate the cut-off values for upregulation (+2) or downregulation (−2). The asterisk (*) indicates where the *P-*value is smaller than or equal to 0.05 (statistically significant results). Standard deviation from the mean fold changes are indicated by the error bars.

Only five genes were significantly upregulated and one downregulated in the liver of recNP-immunized mice, compared to 34 upregulated and nine downregulated in non-immunized adjuvant control mice, and 37 upregulated and eight downregulated in non-immunized PBS control mice (Table S1). There was an almost 100% overlap between the up- or downregulated genes in liver of non-immunized adjuvant and PBS control mice, with only a few genes not consistent between these two groups, most likely as a result of the fold change in some of these genes being on the borderline of the cut-off fold-change value, indicating that mock immunization with the adjuvant alone likely had no effect on immune responses after RVFV challenge. The expression of all 84 genes remained unaltered in the spleen of recNP immunized mice compared to normal mice, whereas nine genes were significantly upregulated and 20 downregulated in adjuvant control mice spleens, compared to 11 upregulated and 20 downregulated in PBS control mice spleens. Comparing tissues, only 10 genes were similarly up- or downregulated in liver and spleen of adjuvant control mice, whereas 45 genes yielded contrasting results between tissues. Only 13 genes were similarly expressed in liver and spleen of PBS control mice, compared to 44 genes which yielded contrasting results between tissues (Table S1), indicating that there was a tissue specific modulation of host gene expression in response to RVFV infection. Selected genes that were significantly up- or downregulated in any of the experimental groups and indicating a clear difference or interesting similarity between immunized and control mice were grouped according to known effects on specific immune functions and shown in Figure 4 (a–b), Figure 5 (a–f) and Figure 6 (a–d).

There was a significant upregulation of several genes in the liver that have pro-apoptotic effects [34]–[42] in non-immunized adjuvant and PBS control mice, whereas these genes were normally expressed in recNP-immunized mice (Figure 4a, Figure 5a). One gene in particular, encoding the early growth response 1 protein (Egr-1) which is a transcription factor involved in proliferation, differentiation and activation of cell death pathways [43], that was upregulated in non-immunized mice was downregulated in recNP-immunized mice. Only one gene encoding the transcription factor RelB, part of the NF-KB family of proteins and responsible for counter-regulating the effects of NF-KB, was upregulated in liver of immunized and non-immunized mice (Figure 5a) [40]. Several genes with anti-apoptotic effects [44]–[49] were downregulated in the liver of non-immunized control mice but normal in recNP-immunized mice (Figure 5b). Most notable of these were the genes encoding the prolactin receptor (Prlr), an anti-inflammatory protein known to promote proliferation, protect against apoptosis and enhance cell survival, and the WW domain containing E3 ubiquitin protein ligase 1 (Wwp1), an anti-apoptotic protein playing a role in proliferation [49]. There was also evidence of severe liver inflammation [43], [50]–[58] in non-immunized adjuvant and PBS control mice, but not in recNP immunized mice (Figure 5c). Despite this the gene encoding the anti-inflammatory cytokine IL-10 was upregulated in all mice. The expression of osteopontin (gene Spp1), important for wound healing, was upregulated significantly in liver of non-immunized control mice, compared to normal expression in immunized mice (Table S1) [58], [59]. The gene expressing the Cyclin-dependent kinase inhibitor P21 (Cdkn1a), a protein with pro- or anti-apoptotic effects and normally upregulated in response to liver injury, was upregulated in liver of all mice but more pronounced in non-immunized mice (Table S1) [60]. The gene expressing the inducible nitric oxide synthase (Nos2), an effector of the innate immune system targeting viral proteases and inhibiting viral replication, was normal in liver of recNP-immunized mice but upregulated in non-immunized mice. Nitric oxide, a product of the Nos2 gene, is a radical molecule that can become toxic under oxidative stress conditions (Table S1) [61].

Both arms of the adaptive immune response, humoral (Th2) and cellular (Th1) [45], [50], [52], [62]–[74], were activated in liver of non-immunized control mice, but normal in immunized mice, at 72 hours. The gene encoding the Phosphatidylinositol 3-kinase catalytic delta polypeptide (Pik3cd), involved in the regulation of B-cells and antibody production, was also upregulated in liver of recNP-immunized mice indicating re-activation of humoral immunity in mice recognizing one of the viral proteins as one it has encountered before (Table S1) [75]. The genes encoding the Dedicator of cyto-kinesis 2 (Dock2) protein and interleukin-12b, which are involved in the development and induction of NKT cells, were upregulated in liver of non-immunized control mice. Some important genes were, however, downregulated in liver of non-immunized control mice (Figure 5 d and Table S1). The gene encoding the Cd1d1 antigen, which is important for the presentation of antigens to, and activation of NKT cells, was downregulated with statistical significance in non-immunized control mice (Figure 5 d) [65], [76], [77]. The expression of the gene encoding interleukin-7, necessary for B- and T-cell and NK cell survival, was downregulated in liver of non-immunized PBS control mice (Figure 5b) [78]. The expression of surfactant protein D (Sftpd), a member of the collectin family, important role player in innate immunity and inhibitor of T lymphocyte proliferation, was upregulated in liver of non-immunized control mice (Figure 5 d) [79]. The expression of the gene encoding the E3 Ubiquitin Ligase Cbl-b, capable of negatively regulating T-cell activation, was upregulated in liver of non-immunized control mice (Figure 5 d) [67], [68], [80]. The expression of the gene encoding interleukin-18, responsible for biasing immunity towards Th-1 cellular immunity and enhancing T-cell cytotoxicity, was downregulated in liver of non-immunized control mice (Figure 5 d) [81]. The expression of the suppressor of cytokine signaling 5 (Socs5), part of a family of proteins that negatively regulate cytokine signaling [82], was upregulated in liver of non-immunized control mice and the gene encoding the immunoglobulin binding protein 1 (Igbp1), a component of receptor cell signaling in B- and T-cells [45], was downregulated in liver of non-immunized control mice. Other genes (Cd81 and Pik3r1) involved in activation, signaling and differentiation of B- and T-cells were also downregulated in liver of non-immunized control mice (Table S1) [78], [83].

An important role player in innate immunity, NK cells, was also activated in liver of non-immunized control mice (Figure 5 f) [64], [84], [85]. The gene encoding the histocompatibility 60 A protein, a ligand for an activating receptor on NK cells [85], was upregulated in non-immunized control mice. However, expression of two important genes in NK cell activation and maturation, IL-18 and the prolactin receptor (Prlr), were downregulated in liver of non-immunized control mice [81], [84]. The expression of three genes encoding Toll-like receptors, a component of the innate immune system responsible for recognizing conserved structures of pathogens, were analyzed with only Tlr4 being upregulated in liver of non-immunized control mice (Table S1) [86].

In contrast to the liver, there was not such a clear bias towards apoptosis in the spleens of adjuvant and PBS control mice (Figure 4b, Figure 6a). Only two genes with pro-apoptotic effects were upregulated, and two genes with anti-apoptotic effects downregulated in spleen of control mice. Interestingly expression of Nos2 and Cd40, both with known pro-apoptotic effects and upregulated in liver of control mice, were downregulated in spleen of control mice. Similarly to liver, some genes with pro-inflammatory effects were upregulated in the spleens of adjuvant and PBS control mice (Figure 6b), except for the genes encoding Nos2, Cd40 and Cd40 ligand. The expression of various genes involved in the activation of B- and T-cell immunity were, however, downregulated in the spleens of adjuvant and PBS control mice (Figure 6c–d). The most notable of these were Cd40 and Cd40 ligand which are responsible for inducing effective CD8+ cytotoxic T-lymphocyte (CTL) responses and activation of B-cells for antibody production [51], and Cd8a and Cd8b1 which are responsible for CTL activation (Figure 6c) [87]. Expression of Complement receptor 2 (Cr2), which plays an important role in B-cell activation and maturation, was also downregulated in spleen of non-immunized control mice (Figure 6d).

## Discussion

The immune evasion mechanisms known for RVFV are directed against the type I interferon response [24], [28], [88] and programmed cell death [29]. However, it was recently shown that mice displaying an earlier and stronger type I interferon response were less susceptible to RVFV infection than mice with a delayed and partial response which suggests some additional evasive or regulatory effects on innate or adaptive immune mechanisms enabling its replication [33].

Immunization of mice with recNP combined with Alhydrogel protected against disease and significantly reduced viral replication [17]. A study by Lorenzo et al (2008) implicated a cellular (Th-1) response to the RVFV NP as a protective mechanism [12], however, our present data shows that immunization with recNP/Alhydrogel induces Th-2 humoral immunity. The discrepant outcome of the two studies might be explained by the fact that Lorenzo et al (2008) used DNA vaccination [12], whereas in our study a subunit antigen combined with adjuvant known to favour Th-2 immunity, was used. Immunized mice launched an earlier and stronger type I interferon response compared to non-immunized mice which showed activation of several genes with pro-apoptotic and pro-inflammatory effects, and suppression of anti-apoptotic genes during acute phase of RVFV infection in the liver. This possibly contributed to hepatic damage, which is the main pathological feature of RVF [89]. The expression of several genes involved in the activation and function of NK cells and B- and T-lymphocytes were suppressed in infected mice, indicating additional immune evasion tactics of RVFV.

The induction of expression of IFNβ has been shown to occur *in vitro* at 3–6 hours p.i [33], while in the livers and spleens of immunized mice in this study it occurred at 3 hours p.i. Taking into account that after host infection, the virus is likely conveyed to the lymph nodes where it first replicates before it can spread to the liver and other organs [2], our results show the ability of RVFV to rapidly spread in the infected host. The expression of the same gene was, however, not upregulated in the tissues of adjuvant and PBS control mice early after infection. Type I interferon could not be upregulated as a direct result of recNP immunization since it is not memory dependent. The fact, however, that the protective anti-recNP responses shown here was largely humoral, but without neutralizing effects, suggests that some other form of antibody dependent mechanism, such as antibody-dependent cell-mediated cytotoxicity (ADCC) or complement-dependent cytotoxicity (CDC), might be responsible. It appears that the RVFV nucleocapsid protein might be released from infected cells independently from other viral proteins [13], and/or processed by the infected host cell proteasome into peptides that are displayed on their surface by MHC-1. Consequently these cells can be coated with anti-NP IgG antibodies complexed with presented NP or peptides, and become a target for ADCC or CDC. It has been shown for influenza virus, with highly variable envelope glycoprotein antigens but a conserved internal nucleoprotein, that previous infection causes heterosubtypic immunity and that this is due to antibodies against the NP involving CD8+ cells in an antibody dependent manner [90]. The resultant lysis of RVFV infected cells, that would otherwise have evaded the host innate immune response and produced progeny virus because of the inhibitory action of NSs on type I interferon might have resulted in the activation of the type I interferon response in neighbouring uninfected cells. The lysed cells would release dsRNA, a by-product of viral replication and activator of type I interferon, helping close-by uninfected cells to remain uninfected and thus decrease virus spread [91]. It has been shown that an early type I interferon response is protective against RVFV infection [18], [33] and it is thus highly likely that the early expression of IFNβ in immunized mice contributed to effective viral clearance and protection. The excessive expression of IFNβ, however, in control mice later after infection (72–120 h) was not able to curb the replication of the virus, as evidenced by high viral titers, and probably contributed to the pathology in the liver and spleen. It has been suggested that the role of IFNγ in RVFV pathogenesis is negligible [28] yet it has been shown *in vivo* that IFNγ does act as an antiviral against RVFV [27]. In our study expression of IFNγ was upregulated in all mice after infection, and there was only a decrease in viral replication in immunized mice indicating that IFNγ did not play a significant role in protection. Also, the massive expression of IFNγ in control mice late after infection did not contain viral replication in the liver and likely rather contributed to liver pathology.

The improper expression of IL-10 during a viral infection might contribute to immune escape events since IL-10 is an anti-inflammatory and immunosuppressive cytokine that inhibits the actions of Th-1 and NK cells, decreases antigen presentation and limits the production of various important cytokines (i.e. IL-12, IL-18 and TNF-α) [73]. It has been shown that IL-10 is upregulated after West Nile virus (WNV) infection, and that IL-10 deficient mice are less susceptible to WNV infection than mice expressing the gene normally [92]. Dengue virus has also been shown to replicate less efficiently when IL-10 expression is suppressed [93]. Some viruses even express IL-10 homologs to enable them to modulate the host immune system and escape viral clearance [94]–[96]. There was an early upregulation of IL-10 expression in liver of adjuvant control mice when an anti-inflammatory response would seem unnecessary. The early detection of replicating RVFV in liver of adjuvant control mice might be contributed to immune escape because of this early activation of IL-10. During the later stages of infection when high level virus replication was noted in non-immunized mice, the host upregulated expression of IL-10 to counteract severe inflammation of tissues. A consequence of this, however, is the suppression of various immune responses as described above which possibly contributed to inefficient clearance of virus in control mice or immune escape. The cytokines IL-10 and IFNγ are counter-regulatory of each other with IFNγ being responsible for pathogen clearance and IL-10 minimizing pathology [73], and therefore their relative expression levels can be an indication of host immune response bias towards virus clearance or minimizing immunopathology. At the acute phase of infection and height of viral replication in non-immunized mice the IL-10 to IFNγ ratio was massively favoured towards IL-10, indicating that these host responses were biased towards decreasing inflammation and pathology rather than clearing the virus, which most likely lead to ineffective viral clearance and high virus titers still being detectable at 120 hours p.i. It was interesting to note replication of virus to a much higher titer in spleen of recNP immunized mice compared to liver. This, however, did not result in any form of clinical disease in immunized mice which is further evidence that early disease progression is proportional to viral replication in the host liver and not necessarily other organs.

The activation of genes with pro-apoptotic and pro-inflammatory effects and the suppression of genes with anti-apoptotic effects in the liver of control mice as a result of uncontrolled viral replication, most probably contributed to severe hepatic disease. In the spleen activation of apoptosis and inflammation was not as pronounced as in the liver of non-immunized mice at 72 hours p.i. The liver is, however, the main target organ for viral replication, the primary site for lesions and rapid severe hepatic damage is probably responsible for the early clinical signs of RVF [89]. The overexpression of CD40, a member of the TNF receptor superfamily and potent activator of nuclear factor kappa beta, in the liver of non-immunized mice is of particular importance to apoptosis and inflammation. Mice deficient in the expression of CD40 have been shown to have improved survival during bacterial sepsis as a result of decreased induction of IL-6, IL-10, IL-12 and IFNγ expression [97]. Contrary to upregulation in liver the expression of CD40, as well as its ligand CD40L (CD154), was downregulated in spleen of non-immunized mice during acute infection. Apart from its role in apoptosis of hepatocytes, CD40 plays a very important role in mediating B- and T-cell responses by controlling cytokine secretion, proliferation and differentiation of B- and T-cells [51]. In addition to downregulation of CD40, various other genes involved in B- and T-cell immunity were also downregulated in the spleens of non-immunized mice. These include NOS2, CD3e, IL7, IL18, CD8a,CD1D1, CD74, CR2 and IL4. The spleen is a very important organ in the host immune system and these results indicate that infection with RVFV has a marked effect on the regulation of the host immune response on a molecular level. It is possible that this broad downregulation of humoral and cellular immunity could have resulted in uncontrolled replication of RVFV, since the virus was still detectable at high titers 2 days later in non-immunized mice. The expression of a TNF receptor superfamily ligand (Tnfsf14) was also upregulated in liver of control mice, but normal in their spleens. This protein is able to block TNFα-mediated apoptosis but not FAS-mediated apoptosis, and is a co-stimulatory factor that enhances T-cell-mediated immunity, leading to severe inflammation [41], [98]. The upregulation of the genes expressing the cell survival factor osteopontin (Spp1) and the Cyclin-dependent kinase inhibitor P21 (Cdkn1a), which is upregulated in response to tissue injury [99], [100], is evidence of the host's attempt to counteract the damaging effects of the infection. P21 interacts, amongst others, with the growth arrest and DNA damage-inducible gene 45 (Gadd45) [101], which was also upregulated in control mice. Gadd45 has been implicated in DNA repair, apoptosis, regulation of signal transduction and cell cycle control [102]. The NSs protein of RVFV has been shown to interact with some specific regions of host cell DNA, causing defects in host chromosome structure and segregation [103]. Therefore it might be that these DNA damage inducible proteins are upregulated in an attempt to arrest the cell cycle of affected cells and prevent apoptosis. The fact that P21 was upregulated in healthy recNP-immunized mice is probably as a result of the very low level of viral replication in their livers.

Although the expression of some genes were similarly regulated during acute infection in the livers and spleens of non-immunized mice, a number of genes were quite distinctly only up- or downregulated in either liver or spleen, with some even being upregulated in one tissue and downregulated in the other. This distinct expression pattern between tissues is very likely a result of the different cellular composition of the liver and spleen. The liver consists mostly of hepatocytes, whereas the spleen consists of a red pulp where red blood cells are filtered and monocytes are stored, and a white pulp which consists of B- and T-lymphocytes. It seems like the specific pattern of genes regulated during acute infection in the liver actually contributed to liver pathology by enhancing apoptosis and inflammation without being able to decrease viral replication to less virulent levels. On the other hand in the spleen the expression of genes involved in activation and modulation of B- and T-cell immunity were altered during acute infection, probably contributing to persistence of the virus in the host up to 120 hours. It was very interesting to note that, despite viral replication to a relatively high level in spleen of recNP immunized mice at 72 hours p.i., none of the 84 genes analyzed were up- or downregulated in their spleens. The mechanism by which recNP immunized mice can, 72 hours after infection, maintain expression levels of genes involved in immune activation similar to expression levels in spleens of uninfected mice, despite viral replication close to that in non-immunized mice where several genes were altered, needs to be further investigated. It is, however, fair to assume that this normal expression of genes involved in immune activation at 72 hours p.i. contributed to efficient viral clearance in immunized mice spleens, as is evidenced by the absence of replicating virus at the next sampling point (120 h). Further it is also fair to assume that the downregulation of B- and T-cell immunity in non-immunized mice at 72 hours p.i. contributed to the virus still replicating in their spleens at 120 hours p.i. Therefore, although not the main target organ of RVFV and not as important for disease progression as the liver, the spleen seems to be very important in regulating immune responses and decreasing the replication of the virus with dysregulation resulting in immune evasion. Further evidence of the dysregulation of host immune responses by RVFV infection is the fact that up- or downregulation of several genes (i.e. IL-18, IL-12b, CD40 and SOCS5) indicate bias towards both Th1 and Th2 immunity in the same host.

In conclusion, the expression of type I IFN was upregulated in the liver and spleen of immunized mice shortly after RVFV challenge, compared to a delayed upregulation of the same gene in non-immunized mice. In the acute phase of liver infection, however, there was a massive upregulation of type I and II interferon in the presence of high viral titers in non-immunized mice associated with downregulation of several genes involved in the activation of B- and T-cells in the spleen, compared to normal expression in immunized mice. Furthermore various genes with pro-apoptotic and pro-inflammatory effects were strongly upregulated, and anti-apoptotic genes downregulated in liver of non-immunized mice.

Host gene responses identified in this study may be useful targets for the development of therapeutic interventions, e.g. suppressing inflammatory and apoptotic effects of RVFV infection in the liver, or limiting the suppression of B- and T-cell activation in the spleen, and further aid the evaluation of subunit candidate vaccines.

## Materials and Methods

### Ethics statement

This study was carried out in strict accordance with the recommendations of the South African National Standards for the Care and Use of Animals for Scientific Purposes (SANS 10386:2008) and the Guidelines for the Use and Care of Animals in Experimental Education and other Scientific Purposes of the University of the Witwatersrand, South Africa. The protocol was approved by the University of the Witwatersrand Animal Ethics Screening Committee (clearance certificate number AESC 2008/16/4). Blood collection after immunization was done under Ketamine/Xylazine anaesthesia, organ tissue collection after viral challenge was done post-mortem after euthanasia with carbon dioxide (CO_2_) asphyxiation, and all efforts were made to minimize suffering.

### Bacterial expression of recombinant RVFV N protein

Bacterial expression and purification of the recombinant N protein was carried out as described previously [10].

### Mouse immunization

Four-week old female BALB/cOlaHsd (Harlan Laboratories, U.K LTD) mice were housed in groups of six in standard plastic mouse cages with wood shaving bedding in a specific pathogen free (SPF) facility during the immunization part of the experiment. Group sizes were calculated so as to have three mice per group per time point for each collection to enable statistical relevance. The immunized group consisted of 60 mice each immunized with a 200 µl inoculum containing 70 µg RVFV recNP in combination with Alhydrogel (Sigma, U.S.A). The adjuvant control group consisted of 18 mice inoculated with Alhydrogel in PBS. The placebo control group consisted of 18 mice which were inoculated with PBS buffer. The normal control group consisted of 18 mice that did not receive any inoculation during the immunization experiment, but were included as an age related control group for the gene expression experiment. All mice, except the age-related normal control group, were inoculated subcutaneously (s.c.) and received identical booster inoculums on day 14 after the initial inoculation. Three mice from the recNP-immunized group were anaesthetized by intramuscular inoculation with a combination of Ketamine (35 mg/kg body mass) and Xylazine (5 mg/kg body mass) and bled by cardiac puncture on each of the following days post-immunization to monitor development of immune responses: day 0, 3, 5, 7, 10 and 12 after the first immunization, and day 0, 3, 5, 7, 12, 18, 21 and 27 after the booster immunization.

### Monitoring of immune response

Anti-recNP humoral responses were monitored by the indirect ELISA which was done as described previously [17]. A mouse serum with high levels of both IgG1 and IgG2A subclass antibodies, generated by immunization with RVFV recNP combined with SaponinQ adjuvant (Sigma, U.S.A) in a previous study [17], was used as a positive control. The positive control serum yielded similar optical density values at 405 nm for IgG (1.094), IgG1 (1.01) and IgG2A (0.94) subclass antibodies at the same dilution used for experimental sera (1∶400). Optical density (OD) was determined at 405 nm for each IgG subclass and means calculated for duplicate measurements of each sample or control. The mean OD values of samples for different IgG subclass antibodies were converted to percentage positivity (PP) values by the following calculation: PP = Mean OD sample/Mean OD positive control ×100, using the OD values described above for IgG, IgG1 and IgG2A to calculate PP values for those specific subclasses. The PP values determined for each of the three biological replicates at each time point (three mice sampled per time point) were then used to calculate the biological mean PP values and standard deviations (S.D.) at each sampling point for the different IgG subclasses.

### RVFV challenge

During the RVFV challenge phase of the experiment the mice were housed as during the immunization part except for being moved to a Biosafety level 3 animal facility.

Vero cells were cultivated in Eagles Minimal Essential Medium (EMEM) (BioWhitaker, MD, U.S.A) containing L-Glutamine, non-essential amino acids, antibiotics (100 IU penicillin, 100 µg streptomycin and 0.25 µg amphotericin B) and 10% foetal bovine serum (Gibco) and maintained at 37°C in 5% CO_2_ incubator. The SPU22/118 KEN 07 strain of RVFV was isolated from a RVF human case during the 2007 Kenyan epidemic [104].

Second passage of the virus, propagated in Vero cells, was used for the challenge of mice on day 28 after the booster immunization. The recNP immunized, adjuvant control and PBS control mice were inoculated s.c. with 100 µl of 10^7.0^ TCID_50_/ml RVFV, whereas the age related control group of mice were “mock” challenged with 100 µl uninfected Vero cell culture supernatant. Mice were monitored twice daily for clinical signs.

Three mice were randomly collected from each group, euthanized by CO_2_ asphyxiation and liver and spleen tissues collected at the following time points after RVFV challenge: 3, 6, 12, 24, 72 and 120 hours. A piece of approximately 50 mg of each tissue was collected into an RNA stabilization reagent (RNAlater, Qiagen, Germany) according to the instructions of the manufacturer, and stored at −70°C until further processing. The remaining tissue was stored in cryotubes at −70°C for viral load determination.

### Virus titrations

Liver and spleen tissues were homogenized as 10% (w/v) suspensions in EMEM containing L-glutamine, non-essential amino acids and antibiotics (100 IU penicillin, 100 µg streptomycin and 0.25 µg amphotericin B) using a Tissuelyser II and 5 mm stainless steel beads (Qiagen, Germany) according to the manufacturer's instructions (4 min, 25 Hz). After centrifugation at 10,000× g for 3 minutes the supernatants were collected and stored at −70°C until tested. Titrations were performed as described previously [17]. Virus titers, calculated by the Kärber method [105], were expressed as median tissue culture infectious dose (TCID_50_) per gram of tissue. Means and standard deviations from the means were determined based on three animals per group per each time point tested.

### RNA extraction

The tissues were moved from RNAlater into lysis buffer (Buffer RLT, Qiagen, Germany) and lysis-homogenization performed by using a Tissuelyser II as described above. Lysates were centrifuged for 3 minutes at 13,200 rpm at room temperature and supernatants collected. Extraction of RNA from the supernatants was performed by using the RNeasy Mini Kit (Qiagen, Germany). A 50% ethanol was used to increase RNA yield from livers and 70% ethanol for spleens as suggested by the manufacturer, followed by on-column Dnase digestion using the Rnase-free Dnase set (Qiagen, Germany), to remove genomic DNA. The RNA was eluted in Rnase-free water, the concentration determined using a NanoDrop ND-1000 spectrophotometer (Thermo Scientific, U.S.A) and stored at −70°C until further testing.

For testing with the SABiosciences quantitative PCR array (as described below), further RNA cleanup was performed on RNA already extracted from livers and spleens collected at 72 hours post infection, as per the manufacturer's instructions. This cleanup, which introduces an additional Dnase digestion, was performed using the RT^2^ qPCR-Grade RNA Isolation Kit (SABiosciences, Qiagen, U.S.A) as per the manufacturer's instructions. The RNA was eluted in Rnase-free water, the concentration determined using a NanoDrop ND-1000 spectrophotometer (Thermo Scientific, U.S.A) and stored at −70°C until further testing.

### Quantitect qRT-PCR

The RNA extracted from all experimental mice at all time points after RVFV challenge were diluted to 10 ng/µl and used in the qRT-PCR reactions. The Quantitect qRT-PCR reactions were performed as described by the manufacturer (Quantifast SYBR Green RT-PCR kit, Qiagen, Germany) using a Lightcycler 1.5 (Roche, Germany). Briefly, a reaction mix was prepared by mixing 2× Quantifast SYBR Green RT-PCR Master Mix (HotStarTaq Plus DNA Polymerase, Quantifast SYBR Green RT-PCR buffer, dNTP mix and ROX passive reference dye), 10× Quantitect Primer set (for detection of the genes for Gapdh, Il10, Ifng and Ifnb1), Quantifast RT-Mix (Omniscript RT and Sensiscript RT), template RNA (10 ng) and RNase-free water to a final volume of 20 µl per reaction. This mix was transferred to 20 µl Lightcycler Capillaries (Roche, Germany) and run on the Lightcycler 1.5 using the following cycles: 1× reverse transcription (10 min, 50°C), 1× hotstart PCR activation (5 min, 95°C) and 40× cycles of denaturation (10 sec, 95°C) and annealing/extension (30 sec, 60°C), with fluorescence data collection just after the annealing/extension step. The threshold cycle (C_T_) values were determined using the second derivative maximum method (Lightcycler Data Analysis Software version 3.5.28, Roche). The C_T_ values were then used for relative quantification (as described below).

RNA was extracted from the liver and spleen of a normal BALB/cOlaHsd mouse to generate standard curves in order to determine PCR reaction efficiencies (necessary for the relative quantification calculations) with primer sets for different genes (GAPDH, IL-10, IFNã and IFNâ1). Dilution series were prepared and the following amounts of RNA tested in duplicate using all primer sets: 30 ng; 15 ng, 7.5 ng, 3.75 ng and 1.875 ng. The C_T_ values and their corresponding template amount values were then used to determine the PCR reaction efficiencies using the Relative Expression Software Tool (REST, Qiagen, Germany) [106] as described by the manufacturer (results not shown).

### Relative quantification data analysis (Quantitect qRT-PCR)

Threshold values (C_T_) from biological triplicates for the different genes analyzed as determined for the immunized mice, adjuvant control and PBS control mice were first normalized to the C_T_ values of the housekeeping gene (Gapdh) analyzed in the same samples, and further normalized to the C_T_ values from the non-infected control mice to determine the relative changes in gene expression compared to age related normal mice. This is the so-called 2^−ΔΔCt^ and the result is a fold change value that is an indication of the expression level of a gene in the experimental group being higher or lower than the expression level in the normal age-related control group [107]. These calculations were done using the REST software (Qiagen, Germany) which uses the PCR efficiencies for different primer sets (as determined above, results not shown) and the C_T_ values of the biological triplicates. The negative inverted values were determined (i.e. 0.5 = ^1^/_−0.5_ = −2.0) for fold change values smaller than one (<1). Genes were only regarded as upregulated with fold changes ≥2.0, and downregulated with fold changes ≤−2.0, based on the same cut-off values used in a recent study [33].

### SABioscienes quantitative PCR array

The RNA extracted with the RT^2^ qPCR-Grade RNA Isolation Kit from liver and spleen collections of all mice at 72 hours post infection were diluted to 150 ng/µl in nuclease free water. Complementary DNA (cDNA) was prepared from the RNA using the RT^2^ First Strand kit (SABiosciences, Qiagen, U.S.A) as described by the manufacturer. A total of 1.2 µg of each RNA preparation was mixed with 5× genomic DNA Elimination buffer and the reaction incubated at 42°C for 5 minutes (total volume 10 µl). After the incubation, the reactions were immediately moved to ice and, subsequently, an equal volume of RT-coctail mix added (5× RT buffer, primers and external control mix, RT-enzyme mix and RNase-free water). These reactions were then incubated at 42°C for 15 minutes and 95°C for 5 minutes. The resultant cDNA of each preparation was then diluted 1∶10 with nuclease-free water and stored at −20°C until tested.

The 1∶10 diluted cDNA was mixed with the master mix (2× SABiosciences RT^2^ qPCR Master Mix) and nuclease-free water, and aliquoted onto the PCR array plates containing primer sets (listed in Table S1)(25 µl of reaction mix per well)(PAMM-053, SABiosciences, Qiagen, Germany). Plates were run on an ABI 7500 cycler (Applied Biosystems, U.S.A). The following cycling program was used: 1×95°C for 10 minutes, 40×95°C for 15 seconds and 60°C for 1 minute, followed by the default melting curve program. Fluorescence was measured just after the 1 minute/60°C step. The cycle threshold (C_T_) values were determined using the cycler software and an automatic baseline adjustment (ABI 7500 Software Version 2.0.1, Applied Biosystems, U.S.A). Three mice per group were analyzed and average values calculated.

### Relative quantification data analysis (SABioscienes quantitative PCR array)

Calculation of fold change values from the results of the SABiosciences quantitative PCR array relies essentially on the same principles as for the Quantitect qRT-PCR, making use of the 2^−ΔΔCt^ method, except for the use of up to 5 housekeeping genes (Gusb, Hprt1, Hsp90ab1, Gapdh and Actb) for normalization of data. These calculations were done using the SABiosciences PCR Array Data Analysis Template Excel Utility (http://sabiosciences.com/pcrarraydataanalysis.php)(Qiagen, Germany). The negative inverted values were determined (i.e. 0.5 = ^1^/_−0.5_ = −2.0) for fold change values smaller than one (<1). Genes were only regarded as upregulated with fold changes ≥2.0, and downregulated with fold changes ≤−2.0.

### Statistical analysis

The REST software uses a Pair-Wise Fixed Reallocation Randomization Test© [106] with 2000 randomizations to determine a *P*-value which gives an indication of the statistical significance of fold changes. The SABiosciences PCR Array Data Analysis Template Excel Utility incorporates the calculation of a *P*-value using a T-test. Fold changes with *P*-values smaller or equal to 0.05 (≤0.05) were taken as statistically significant. Three biological replicates were used at all experimental time points in all groups to enable statistical reliability of results.

## Supporting Information

Table S1 **Fold change in expression of 84 genes involved in activation of B- and T-cell immunity.** Fold changes are shown for immunized mice versus non-immunized control mice (n = 3 per group) after RVFV challenge at 72 hours in liver, relative to expression in an age-related control group of mice (n = 3).
(DOC)
