# Supplementary material for: Anti-Nucleocapsid Protein Immune Responses Counteract Pathogenic Effects of Rift Valley Fever Virus Infection in Mice
Source: PLoS One. 2011 Sep 16;6(9):e25027. doi: 10.1371/journal.pone.0025027 (PMC3174991; doi:10.1371/journal.pone.0025027)
Supplement: Table S1 — Fold change in expression of 84 genes involved in activation of B- and T-cell immunity. Fold changes are shown for immunized mice versus non-immunized control mice (n = 3 per group) after RVFV challenge at 72 hours in liver, relative to expression in an age-related control group of mice (n = 3). (DOC) [file pone.0025027.s001.doc]

| **Gene** | **Gene name** | **Transcript accession number** | **recNP Immunized**  **Fold change and**  **P-value**  **Liver Spleen** | | **Adjuvant control**  **Fold change and**  **P-value**  **Liver Spleen** | | **PBS control**  **Fold change and**  **P-value**  **Liver Spleen** | |
| --- | --- | --- | --- | --- | --- | --- | --- | --- |
| *Ap3b1* | Adaptor-related protein complex 3, beta 1 subunit | NM_009680 | 1.65  p = 0.21 | -4.41  p = 0.28 | -1.09  p = 0.83 | -1.14  p = 0.21 | 1.13  p = 0.58 | -1.19  p = 0.04 |
| *Bad* | BCL2-associated agonist of cell death | NM_007522 | 1.06  p = 0.62 | -1.14  p = 0.13 | **-2.19***  **p = 0.0004** | -1.48  p = 0.007 | -1.64  p = 0.001 | -1.31  p = 0.03 |
| *Cxcr5* | Chemochine (C-X-C motif) receptor 5 | NM_007551 | 1.56  p = 0.88 | -1.14  p = 0.007 | 5.53  p = 0.18 | -1.33  p = 0.19 | 5.23  p = 0.23 | -1.35  p = 0.07 |
| *Cblb* | E3 Ubiquitin Ligase Casitas B-lineage lymphoma b | NM_001033238 | 1.07  p = 0.68 | -1.10  p = 0.07 | **7.16***  **p = 0.006** | **2.09***  **p = 0.03** | **8.65***  **p = 0.001** | **2.66***  **p = 0.01** |
| *Ccnd3* | Cyclin D3 | NM_007632 | 1.02  p = 0.86 | 1.18  p = 0.09 | 1.12  p = 0.29 | **-3.82***  **p = 0.0002** | 1.27  p = 0.02 | **-4.15***  **p = 0.0001** |
| *Cd1d1* | CD1d1 antigen | NM_007639 | 1.27  p = 0.41 | 1.11  p = 0.11 | **-6.27***  **p = 0.005** | **-2.86***  **p < 0.0001** | **-6.88***  **p = 0.003** | **-2.77***  **p < 0.0001** |
| *Cd2* | CD2 antigen | NM_013486 | 1.02  p = 0.90 | -1.27  p = 0.17 | **2.56***  **p = 0.02** | -1.33  p = 0.14 | **2.66***  **p = 0.04** | -1.03  p = 0.89 |
| *Cd28* | CD28 antigen | NM_007642 | 6.48  p = 0.13 | -1.01  p = 0.84 | **22.33***  **p = 0.002** | -1.14  p = 0.38 | **21.41***  **p = 0.005** | 1.00  p = 0.90 |
| *Cd3d* | CD3 antigen, delta polypeptide | NM_013487 | 1.15  p = 0.23 | 1.02  p = 0.84 | 2.39  p = 0.07 | -1.68  p = 0.007 | 1.86  p = 0.06 | -1.41  p = 0.13 |
| *Cd3e* | CD3 antigen, epsilon polypeptide | NM_007648 | 1.58  p = 0.30 | -1.19  p = 0.03 | 3.44  p = 0.06 | **-2.27***  **p = 0.0005** | **3.28***  **p = 0.02** | **-2.17***  **p = 0.003** |
| *Cd3g* | CD3 antigen, gamma polypeptide | NM_009850 | 1.34  p = 0.20 | -1.21  p = 0.07 | 1.14  p = 0.57 | **-3.15***  **p = 0.0006** | -1.47  p = 0.12 | **-2.86***  **p = 0.003** |
| *Cd4* | CD4 antigen | NM_013488 | 1.06  p = 0.74 | -1.30  p = 0.05 | -1.89  p = 0.45 | **-2.52***  **p < 0.0001** | -1.01  p = 0.92 | **-2.21***  **p = 0.0005** |
| *Cd40* | CD40 antigen | NM_011611 | 2.91  p = 0.06 | -1.42  p = 0.16 | **29.40***  **p = 0.002** | **-2.18***  **p = 0.01** | **25.87***  **p = 0.003** | **-2.66***  **p = 0.004** |
| *Cd40lg* | CD40 ligand | NM_011616 | 1.41  p = 0.48 | -1.44  p = 0.07 | 1.42  p = 0.44 | **-4.30***  **p = 0.004** | 1.83  p = 0.11 | **-4.19***  **p = 0.005** |
| *Cd74* | CD74 antigen | NM_010545 | 2.03  p = 0.12 | -1.46  p = 0.01 | 1.39  p = 0.012 | **-2.87***  **p = 0.003** | 1.36  p = 0.13 | **-3.87***  **p = 0.0004** |
| *Cd81* | CD81 antigen | NM_133655 | 1.04  p = 0.68 | -1.23  p = 0.08 | **-2.17***  **p = 0.002** | -1.57  p = 0.002 | -1.77  p = 0.002 | -1.64  p = 0.002 |
| *Cd8a* | CD8 antigen, alpha chain | NM_001081110 | 2.33  p = 0.40 | 1.05  p = 0.52 | 4.88  p = 0.13 | **-3.05***  **p = 0.001** | 2.81  p = 0.23 | **-3.15***  **p = 0.0007** |
| *Cd8b1* | CD8 antigen, beta chain 1 | NM_009858 | -1.05  p = 0.99 | 1.52  p = 0.42 | 4.08  p = 0.14 | **-3.47***  **p = 0.0003** | 2.39  p = 0.40 | **-3.22***  **p = 0.0009** |
| *Cd93* | CD93 antigen | NM_010740 | 1.14  p = 0.85 | -1.05  p = 0.65 | 83.91  p = 0.06 | **6.64***  **p = 0.005** | **144.67***  **p = 0.001** | **7.23***  **p = 0.0003** |
| *Cdkn1a* | Cyclin-dependent kinase inhibitor 1A (P21) | NM_007669 | **9.02***  **p = 0.02** | 1.70  p = 0.09 | **127.19***  **p < 0.0001** | **13.88***  **p = 0.0002** | **145.01***  **p = 0.0006** | **13.86***  **p < 0.0001** |
| *Clcf1* | Cardiotrophin-like cytokine factor 1 | NM_019952 | 1.27  p = 0.89 | 1.16  p = 0.28 | **33.92***  **p = 0.01** | -1.05  p = 0.74 | **52.10***  **p = 0.002** | 1.12  p = 0.30 |
| *Cr2* | Complement receptor 2 | NM_007758 | -2.0  p = 0.15 | -1.54  p = 0.01 | 1.07  p = 0.74 | **-7.88***  **p = 0.001** | 1.61  p = 0.10 | **-11.58***  **p = 0.0004** |
| *Csf2* | Granulocyte macrophage colony stimulating factor 2 | NM_009969 | -1.56  p = 0.66 | 1.61  p = 0.32 | 2.37  p = 0.45 | **3.88***  **p = 0.05** | 2.09  p = 0.58 | **4.42***  **p = 0.009** |
| *Cxcl12* | Chemokine (C-X-C motif) ligand 12 | NM_021704 | 1.24  p = 0.03 | -1.29  p = 0.05 | **-2.21***  **p = 0.02** | -1.55  p = 0.008 | -1.64  p = 0.008 | -1.26  p = 0.09 |
| *Cxcr4* | Chemokine (C-X-C motif) receptor 4 | NM_009911 | 1.66  p = 0.006 | 1.07  p = 0.37 | **22.12***  **p = 0.01** | **2.61***  **p = 0.004** | **22.84***  **p = 0.002** | **3.02***  **p = 0.0002** |
| *Dock2* | Dedicator of cyto-kinesis 2 | NM_033374 | 1.11  p = 0.43 | -1.22  p = 0.04 | **4.85***  **p = 0.04** | -1.79  p = 0.001 | **3.47***  **p = 0.003** | -1.76  p = 0.005 |
| *Egr1* | Early growth response 1 | NM_007913 | **-5.00***  **p = 0.01** | -1.21  p = 0.16 | **3.88***  **p = 0.005** | 1.37  p = 0.13 | **4.54***  **p = 0.004** | 1.61  p = 0.02 |
| *Flt3* | FMS-like tyrosine kinase 3 | NM_010229 | -1.06  p = 0.87 | -1.52  p = 0.005 | **12.62***  **p = 0.004** | -1.14  p = 0.51 | **9.19***  **p = 0.03** | 1.01  p = 0.87 |
| *Gadd45g* | Growth arrest and DNA-damage-inducible 45 gamma | NM_011817 | 7.26  p = 0.26 | 1.03  p = 0.27 | **5.35***  **p = 0.002** | 1.86  p = 0.0005 | **4.37***  **p = 0.008** | **2.11***  **p = 0.004** |
| *Glmn* | Glomulin, FKBP associated protein | NM_133248 | 1.08  p = 0.59 | -1.07  p = 0.60 | -1.03  p = 0.82 | -1.15  p = 0.35 | 1.05  p = 0.73 | -1.09  p = 0.57 |
| *H2-Aa* | Histocompatibility 2, class II antigen A, alpha | NM_010378 | 1.84  p = 0.11 | 1.32  p = 0.79 | **2.01***  **p = 0.014** | -1.13  p = 0.58 | 1.56  p = 0.16 | -1.60  p = 0.24 |
| *H60a* | Histocompatibility 60a | NM_010400 | -1.51  p = 0.70 | 1.05  p = 0.66 | **16.50***  **p = 0.046** | 1.39  p = 0.16 | **13.99***  **p = 0.036** | 1.53  p = 0.0004 |
| *Hdac5* | Histone deacetylase 5 | NM_010412 | 1.05  p = 0.65 | -1.20  p = 0.19 | -1.46  p = 0.04 | -1.73  p = 0.001 | **-2.18***  **p = 0.02** | -1.95  p = 0.0004 |
| *Hdac7* | Histone deacetylase 7 | NM_019572 | -1.07  p = 0.68 | -1.26  p = 0.02 | **4.83***  **p = 0.012** | -1.73  p = 0.003 | **5.58***  **p = 0.0007** | -1.43  p = 0.006 |
| *Hells* | Helicase, lymphoid specific | NM_008234 | 1.34  p = 0.46 | 1.25  p = 0.10 | 2.15  p = 0.08 | -1.14  p = 0.54 | **2.64***  **p = 0.007** | -1.36  p = 0.05 |
| *Hsp90aa1* | Heat shock protein 90, alpha (cytosolic), class A member 1 | NM_010480 | 1.08  p = 0.67 | 1.23  p = 0.26 | 1.33  p = 0.13 | -1.24  p = 0.04 | 1.48  p = 0.012 | -1.28  p = 0.04 |
| *Icosl* | Icos ligand | NM_015790 | 2.33  p = 0.30 | -1.15  p = 0.05 | 3.48  p = 0.25 | **-2.25***  **p = 0.002** | 3.29  p = 0.07 | **-2.60***  **p = 0.0002** |
| *Ifng* | Interferon gamma | NM_008337 | 4.05  p = 0.32 | 1.61  p = 0.41 | 4.90  p = 0.09 | 1.28  p = 0.44 | 3.10  p = 0.12 | 1.02  p = 0.82 |
| *Igbp1* | Immunoglobulin (CD79A) binding protein 1 | NM_008784 | -1.23  p = 0.15 | -1.14  p = 0.04 | **-2.39***  **p = 0.002** | -1.13  p = 0.13 | **-2.37***  **p = 0.002** | -1.12  p = 0.08 |
| *Igbp1b* | Immunoglobulin (CD79A) binding protein 1b | NM_015777 | 1.39  p = 0.37 | 1.91  p = 0.11 | **7.26***  **p = 0.04** | -1.10  p = 0.78 | 4.53  p = 0.10 | 3.95  p = 0.07 |
| *Il10* | Interleukin 10 | NM_010548 | 3.99  p = 0.21 | -1.05  p = 0.87 | **17.64***  **p = 0.04** | **31.30***  **p = 0.0007** | **10.65***  **p = 0.02** | **34.46***  **p < 0.0001** |
| *Il11* | Interleukin 11 | NM_008350 | 1.93  p = 0.20 | 2.49  p = 0.57 | 275.80  p = 0.13 | 8.35  p = 0.11 | **772.47***  **p = 0.007** | **17.55***  **p = 0.001** |
| *Il12b* | Interleukin 12B | NM_008352 | 2.59  p = 0.25 | 1.48  p = 0.17 | **11.75***  **p = 0.02** | **2.28***  **p = 0.02** | **20.35***  **p = 0.005** | **2.91***  **p = 0.0003** |
| *Il15* | Interleukin 15 | NM_008357 | 1.12  p = 0.48 | 1.81  p = 0.08 | -1.64  p = 0.026 | -1.73  p = 0.004 | -1.54  p = 0.04 | -1.62  p = 0.005 |
| *Il18* | Interleukin 18 | NM_008360 | 1.21  p = 0.12 | 1.09  p = 0.21 | **-3.52***  **p = 0.0002** | **-4.98***  **p = 0.0002** | **-4.91***  **p = 0.0001** | **-7.55***  **p = 0.0001** |
| *Il27* | Interleukin 27 | NM_145636 | 3.25  p = 0.63 | 2.86  p = 0.18 | 5.73  p = 0.32 | **4.57***  **p = 0.03** | 11.42  p = 0.10 | **3.37***  **p = 0.01** |
| *Il2ra* | Interleukin 2 receptor, alpha chain | NM_008367 | 3.18  p = 0.09 | -1.16  p = 0.34 | **5.66***  **p = 0.04** | 1.37  p = 0.14 | 4.41  p = 0.06 | 1.75  p = 0.008 |
| *Il4* | Interleukin 4 | NM_021283 | 2.04  p = 0.35 | 1.01  p = 0.93 | 21.42  p = 0.24 | **-2.18***  **p = 0.05** | **8.73***  **p = 0.03** | **-2.56***  **p = 0.02** |
| *Il7* | Interleukin 7 | NM_008371 | 1.64  p = 0.05 | -1.48  p = 0.10 | -1.70  p = 0.06 | **-3.83***  **p = 0.006** | **-2.38***  **p = 0.009** | **-2.50***  **p = 0.009** |
| *Impdh1* | Inosine 5'-phosphate dehydrogenase 1 | NM_011829 | **2.13***  **p = 0.004** | -1.09  p = 0.33 | 3.26  p = 0.25 | -1.05  p = 0.48 | **4.92***  **p = 0.02** | -1.02  p = 0.72 |
| *Impdh2* | Inosine 5'-phosphate dehydrogenase 2 | NM_011830 | 1.18  p = 0.15 | 1.14  p = 0.02 | 1.00  p = 0.99 | -1.02  p = 0.92 | 1.10  p = 0.48 | -1.11  p = 0.15 |
| *Inha* | Inhibin alpha | NM_010564 | -1.16  p = 0.74 | -1.04  p = 0.90 | 4.49  p = 0.06 | 1.24  p = 0.66 | 3.12  p = 0.17 | 1.18  p = 0.80 |
| *Irf4* | Interferon regulatory factor 4 | NM_013674 | 1.51  p = 0.96 | -1.15  p = 0.09 | 2.59  p = 0.39 | 1.78  p = 0.01 | 1.95  p = 0.79 | 1.72  p = 0.004 |
| *Jag2* | Jagged 2 | NM_010588 | 1.67  p = 0.18 | -1.18  p = 0.34 | **6.79***  **p = 0.02** | 1.51  p = 0.15 | **7.46***  **p = 0.02** | 1.88  p = 0.007 |
| *Ms4a1* | Membrane-spanning 4-domains, subfamily A, member 1 | NM_007641 | 1.39  p = 0.37 | -1.65  p = 0.02 | **7.26***  **p = 0.04** | **-2.88***  **p = 0.01** | 4.53  p = 0.10 | **-3.25***  **p < 0.0001** |
| *Nkx2-3* | NK2 transcription factor related, locus3 | NM_008699 | 1.10  p = 0.72 | -1.20  p = 0.05 | 1.56  p = 0.26 | **-3.13***  **p = 0.0002** | 1.16  p = 0.59 | **-2.69***  **p = 0.001** |
| *Nos2* | Nitric oxide synthase 2, inducible | NM_010927 | 1.05  p = 0.97 | -1.89  p = 0.08 | 25.71  p = 0.06 | **-2.12***  **p = 0.05** | **32.07***  **p = 0.001** | **-5.72***  **p = 0.05** |
| *Pawr* | PRKC, apoptosis, WT1, regulator | NM_054056 | 1.06  p = 0.57 | -1.29  p = 0.04 | **2.68***  **p = 0.0003** | -1.70  p = 0.007 | **3.05***  **p = 0.0006** | -1.26  p = 0.06 |
| *Pdcd1lg2* | Programmed cell death 1 ligand 2 | NM_021396 | 1.90  p = 0.1 | -1.08  p = 0.63 | **12.42***  **p = 0.0004** | 1.18  p = 0.20 | 9.47  p = 0.08 | 1.47  p = 0.06 |
| *Pik3cd* | Phosphatidylinositol 3-kinase catalytic delta polypeptide | NM_008840 | **3.59***  **p = 0.04** | -1.15  p = 0.08 | 5.77  p = 0.13 | -1.32  p = 0.06 | **7.53***  **p = 0.002** | -1.25  p = 0.09 |
| *Pik3r1* | Phosphatidylinositol 3-kinase, regulatory subunit, polypeptide 1 (p85 alpha) | NM_001024955 | 1.10  p = 0.63 | -1.07  p = 0.30 | **-3.80***  **p = 0.007** | -1.99  p = 0.001 | **-3.63***  **p = 0.005** | -1.79  p = 0.003 |
| *Prkcd* | Protein kinase C, delta | NM_011103 | 1.30  p = 0.1 | 1.00  p = 0.94 | **3.73***  **p = 0.03** | -1.02  p = 0.69 | **4.01***  **p = 0.002** | 1.01  p = 0.84 |
| *Prkcq* | Protein kinase C, theta | NM_008859 | 1.92  p = 0.18 | 1.12  p = 0.37 | 4.63  p = 0.09 | -1.95  p = 0.007 | 3.89  p = 0.06 | **-2.18***  **p = 0.004** |
| *Prlr* | Prolactin receptor | NM_011169 | 1.15  p = 0.42 | -1.32  p = 0.13 | **-62.79***  **p = 0.0003** | 1.24  p = 0.4 | **-59.16***  **p = 0.0003** | 1.65  p = 0.02 |
| *Ptprc* | Protein tyrosine phosphatase, receptor type, C | NM_011210 | 2.07  p = 0.57 | -1.05  p = 0.08 | 8.14  p = 0.055 | **-2.01***  **p = 0.0006** | 3.69  p = 0.13 | **-2.08***  **p =** **0.0004** |
| *Rag1* | Recombination activating gene 1 | NM_009019 | -1.05  p = 0.98 | -1.01  p = 0.89 | **4.98***  **p = 0.05** | -7.41  p = 0.09 | 3.11  p = 0.13 | -2.38  p = 0.12 |
| *Relb* | Avian reticuloendotheliosis viral (v-rel) oncogene related B | NM_009046 | **2.50***  **p = 0.04** | -1.09  p = 0.13 | **12.30***  **p = 0.002** | -1.02  p = 0.99 | **15.85***  **p = 0.002** | 1.06  p = 0.57 |
| *Rgs1* | Regulator of G-protein signaling 1 | NM_015811 | 1.67  p = 0.25 | 1.79  p = 0.04 | **41.67***  **p = 0.006** | **3.74***  **p = 0.002** | **24.76***  **p = 0.016** | **3.91***  **p = 0.001** |
| *Sftpd* | Surfactant associated protein D | NM_009160 | 1.30  p = 0.41 | -1.02  p = 0.90 | **20.40***  **p = 0.03** | -2.35  p = 0.21 | **26.35***  **p = 0.0006** | -1.34  p = 0.48 |
| *Sit1* | Suppression inducing transmembrane adaptor 1 | NM_019436 | 1.92  p = 0.24 | -1.26  p = 0.15 | 3.64  p = 0.07 | -1.89  p = 0.007 | 2.96  p = 0.25 | -1.87  p = 0.001 |
| *Sla2* | Src-like-adaptor 2 | NM_029983 | 1.08  p = 0.66 | 1.24  p = 0.10 | 2.38  p = 0.16 | -1.25  p = 0.20 | **2.30***  **p = 0.007** | -1.23  p = 0.18 |
| *Socs5* | Suppressor of cytokine signaling 5 | NM_019654 | -1.01  p = 0.90 | -1.10  p = 0.05 | **2.55***  **p = 0.006** | -1.29  p = 0.03 | **3.63***  **p = 0.0007** | -1.07  p = 0.50 |
| *Spp1* | Secreted phosphoprotein 1 | NM_009263 | -1.10  p = 0.62 | 1.59  p = 0.09 | **29.12***  **p = 0.003** | **27.50***  **p = 0.03** | **26.97***  **p = 0.001** | **26.35***  **p = 0.0006** |
| *Tlr1* | Toll-like receptor 1 | NM_030682 | 1.82  p = 0.2 | -1.19  p = 0.06 | 8.68  p = 0.07 | -1.91  p = 0.003 | 5.55  p = 0.06 | -1.90  p = 0.0005 |
| *Tlr4* | Toll-like receptor 4 | NM_021297 | 3.46  p = 0.06 | -1.06  p = 0.79 | **14.13***  **p = 0.03** | -1.78  p = 0.0005 | **8.28***  **p = 0.005** | -1.74  p = 0.002 |
| *Tlr6* | Toll-like receptor 6 | NM_011604 | 1.30  p = 0.43 | -1.30  p = 0.03 | 2.11  p = 0.14 | -1.23  p = 0.08 | 1.77  p = 0.12 | -1.30  p = 0.025 |
| *Tnfrsf13b* | Tumor necrosis factor receptor superfamily, member 13b | NM_021349 | -1.35  p = 0.76 | -1.48  p = 0.01 | **6.67***  **p = 0.0006** | -1.49  p = 0.06 | 4.27  p = 0.15 | -1.71  p = 0.003 |
| *Tnfrsf13c* | Tumor necrosis factor receptor superfamily, member 13c | NM_028075 | 1.23  p = 0.68 | -1.99  p = 0.01 | 2.88  p = 0.12 | -1.26  p = 0.19 | 2.46  p = 0.15 | -1.26  p = 0.15 |
| *Tnfsf13b* | Tumor necrosis factor (ligand) superfamily, member 13b | NM_033622 | 1.32  p = 0.60 | 1.22  p = 0.05 | **8.74***  **p = 0.014** | -1.30  p = 0.12 | **5.75***  **p = 0.005** | -1.51  p = 0.02 |
| *Tnfsf14* | Tumor necrosis factor (ligand) superfamily, member 14 | NM_019418 | 2.14  p = 0.08 | 1.18  p = 0.22 | **24.66***  **p = 0.04** | 1.07  p = 0.69 | **16.80***  **p = 0.001** | -1.06  p = 0.88 |
| *Traf6* | Tnf receptor-associated factor 6 | NM_009424 | 1.53  p = 0.06 | -1.11  p = 0.34 | 1.72  p = 0.03 | 1.31  p = 0.15 | 2.34  p = 0.06 | 1.37  p = 0.07 |
| *Vav1* | Vav 1 oncogene | NM_011691 | 2.20  p = 0.13 | -1.06  p = 0.29 | **7.50***  **p = 0.03** | -2.03  p = 0.27 | **8.00***  **p = 0.02** | -1.11  p = 0.08 |
| *Was* | Wiskott-Aldrich syndrome homolog (human) | NM_009515 | 1.53  p = 0.50 | -1.20  p = 0.08 | 4.33  p = 0.07 | -1.38  p = 0.008 | **4.64***  **p = 0.004** | -1.50  p = 0.004 |
| *Wwp1* | WW domain containing E3 ubiquitin protein ligase 1 | NM_177327 | -1.07  p = 0.38 | -1.16  p = 0.35 | **-9.16***  **p = 0.0001** | **-3.25***  **p = 0.004** | **-11.99***  **p = 0.0001** | 49.98**†**  p = 0.37 |

**Bold** text and an asterisk * indicates statistically significantly up- or downregulated genes (fold change ≥ 2.0 or ≤ -2.0 and p ≤ 0.05)

**†** Indicates where one of the triplicate qRT-PCR reactions failed resulting in incorrect fold change value. This result is excluded from further discussion.
